# Supplementary material for: Complex‐centric proteome profiling by SEC‐SWATH‐MS
Source: Mol Syst Biol. 2019 Jan 14;15(1):e8438. doi: 10.15252/msb.20188438 (PMC6346213; doi:10.15252/msb.20188438)
Supplement: Supplementary file 8 — Dataset EV7 [file MSB-15-e8438-s008.zip › feature_plots_string/P52815.pdf]

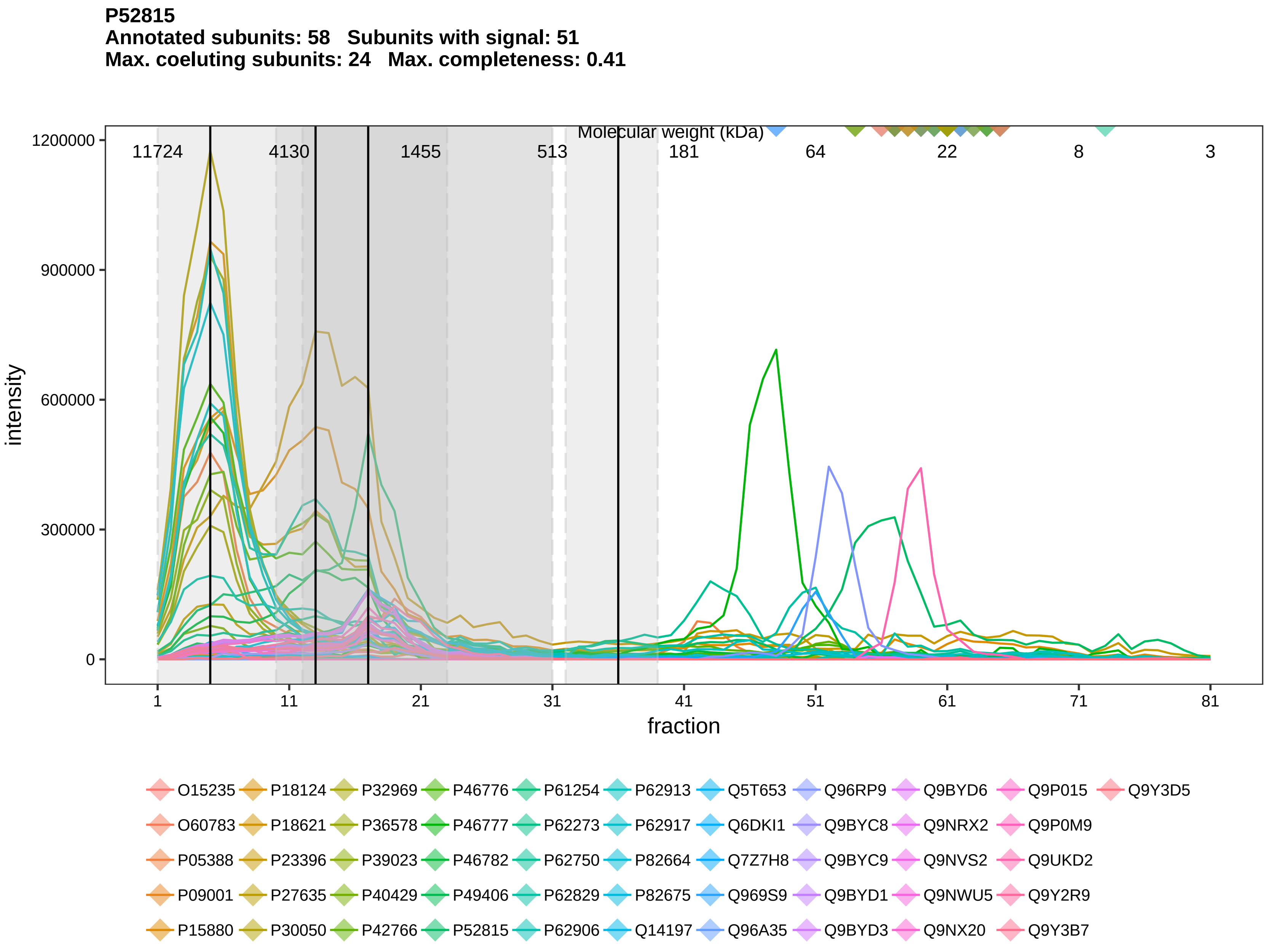
P52815
Annotated subunits: 58 Subunits with signal: 51
Max. coeluting subunits: 24 Max. completeness: 0.41
Molecular weight (kDa)
11724
4130
1455
513
181
64
22
8
3
intensity
1200000
900000
600000
300000
0
fraction
1
11
21
31
41
51
61
71
81
O15235
P18124
P32969
P46776
P61254
P62913
Q5T653
Q96RP9
Q9BYD6
Q9P015
Q9Y3D5
O60783
P18621
P36578
P46777
P62273
P62917
Q6DKI1
Q9BYC8
Q9NRX2
Q9P0M9
P05388
P23396
P39023
P46782
P62750
P82664
Q7Z7H8
Q9BYC9
Q9NVS2
Q9UKD2
P09001
P27635
P40429
P49406
P62829
P82675
Q969S9
Q9BYD1
Q9NWU5
Q9Y2R9
P15880
P30050
P42766
P52815
P62906
Q14197
Q96A35
Q9BYD3
Q9NX20
Q9Y3B7
